# Supplementary material for: The effect of DNA polymorphisms and natural variation on crossover hotspot activity in Arabidopsis hybrids
Source: Nat Commun. 2023 Jan 3;14:33. doi: 10.1038/s41467-022-35722-3 (PMC9810609; doi:10.1038/s41467-022-35722-3)
Supplement: Supplementary file 1 — Supplementary Information [file 41467_2022_35722_MOESM1_ESM.pdf]

The effect of DNA polymorphisms and natural variation on crossover hotspot activity in *Arabidopsis* hybrids

Maja Szymanska-Lejman, Wojciech Dziegielewski, Julia Dluzewska, Nadia Kbiri, Anna Bieluszewska, R. Scott Poethig & Piotr A. Ziolkowski

Supplementary Information

Supplementary Figures

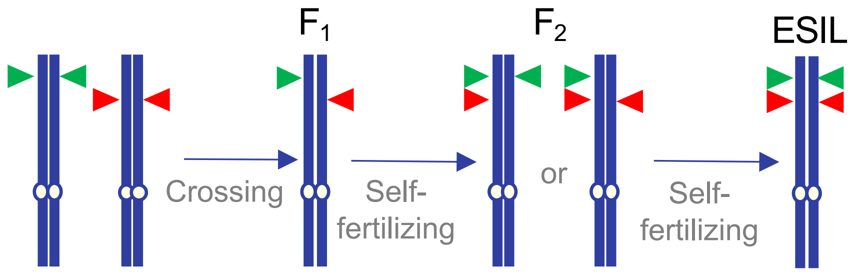

Supplementary Fig. 1. Construction of ESILs. Single-reporter traffic lines were selected based on their location on the same chromosome, crossed together to obtain F_1 plants, and recombinant F_2 seeds carrying two copies of eGFP or dsRed reporter were selected under the stereomicroscope based on fluorescence intensity. The resulting F_2 plants were selfed and seeds homozygous for both reporters were selected as proper ESILs. Locations of reporter cassettes were verified with PCR amplification and Sanger sequencing (not shown).

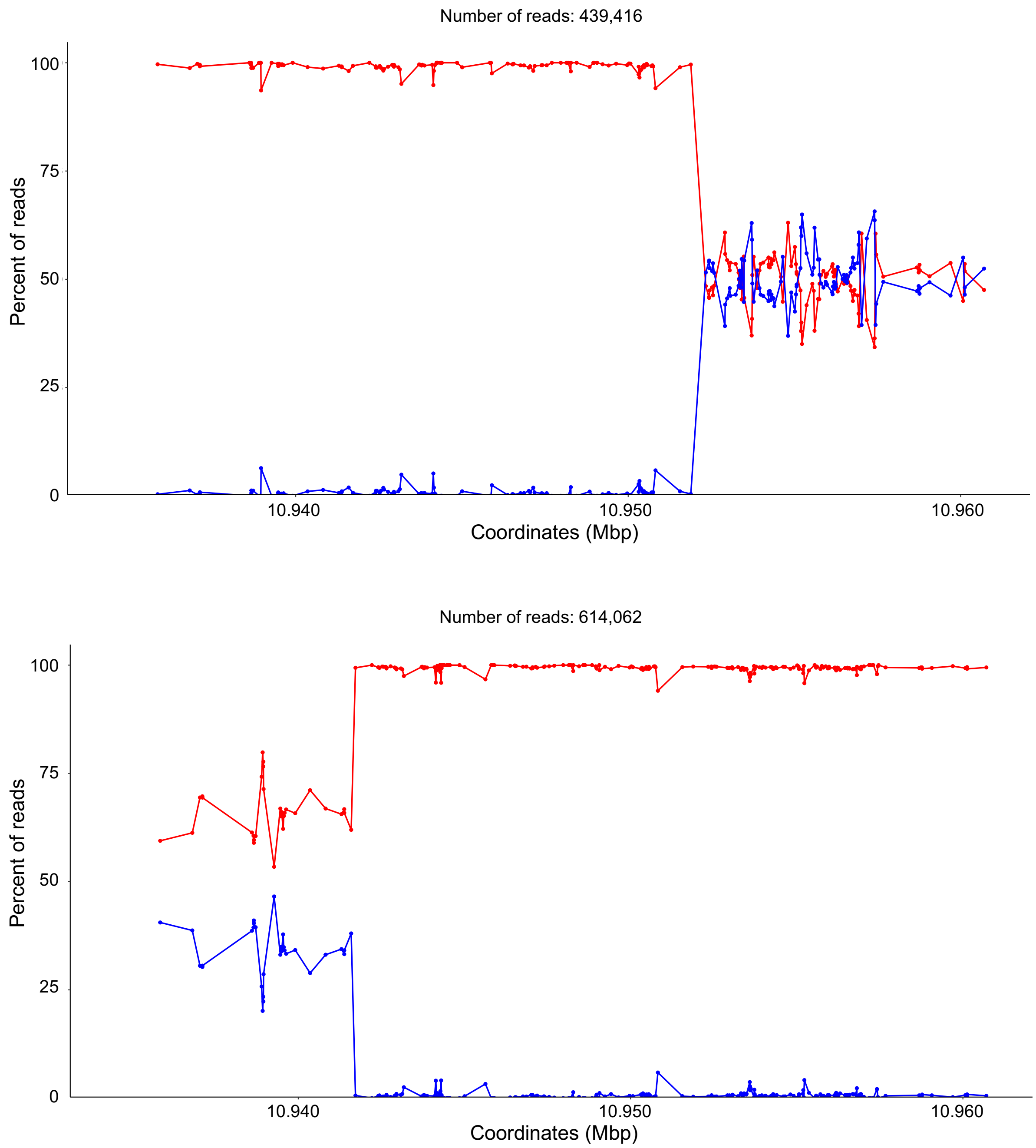

Supplementary Fig. 2. Examples of sequencing results for red (upper panel) and green (lower panel) recombinants shown in Fig. 1E. The x-axes represent the coordinates within the ChP interval, while y-axes show percentage of reads for each SNP assigned as Col (blue dots) or L*er* (red dots). Crossover site is defined as the fragment between two SNPs showing transition of genotype.

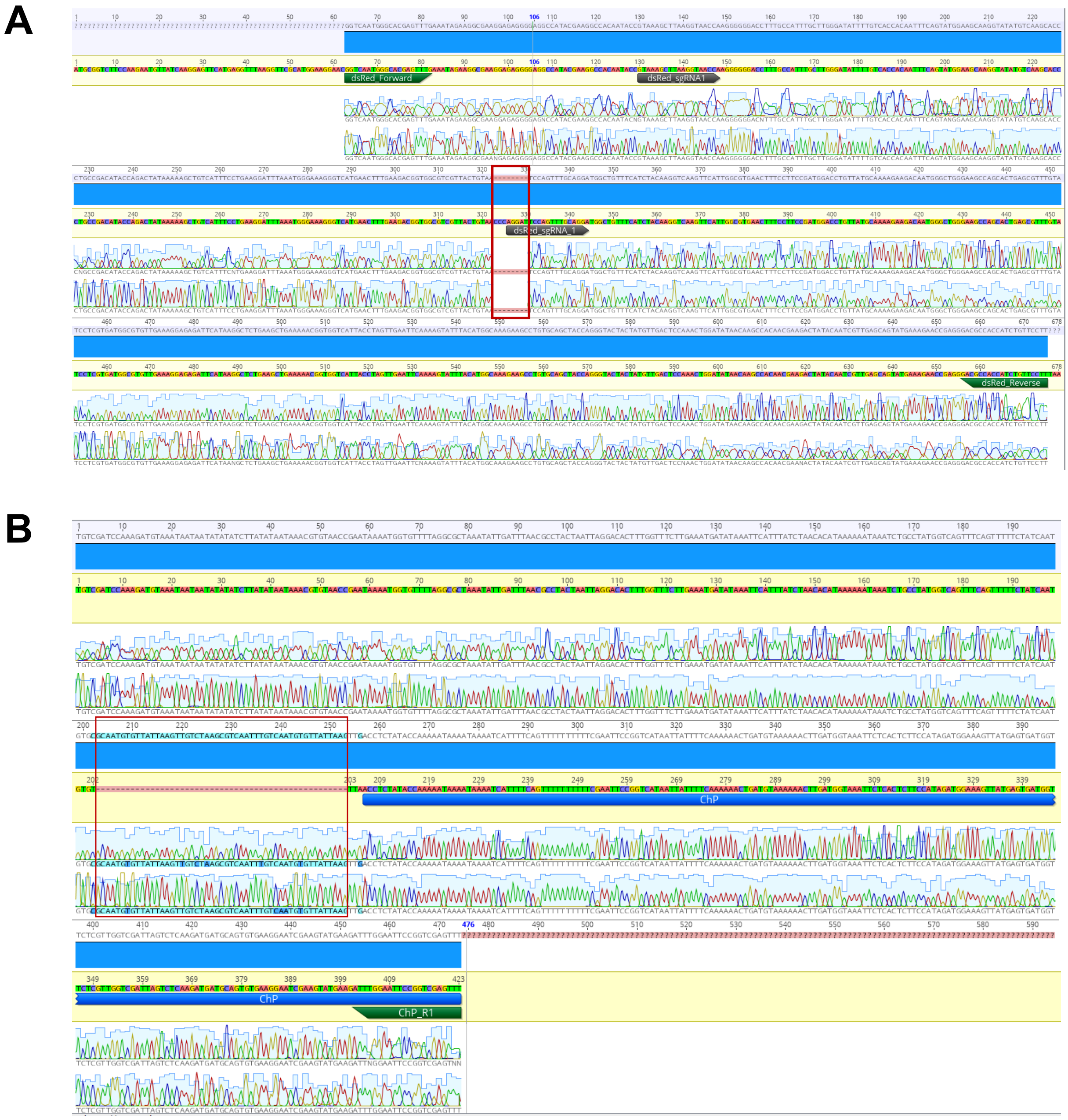

Supplementary Fig. 3. Pseudoreporter line for ChP. **A**, Sequencing results mapped to dsRed coding sequence. Red frame indicates an 8 bp frame-shift mutation resulting in loss of red fluorescence. gRNAs used to generate the pseudoreporter line are marked in gray. Primers used for the amplification are marked in dark green. **B**, Sequencing results mapped to Col-0 reference sequence. Red frame indicates the 51 bp insertion that is a result of Cas9-mediated deletion of the whole eGFP reporter cassette. The beginning of ChP interval is marked in dark blue.

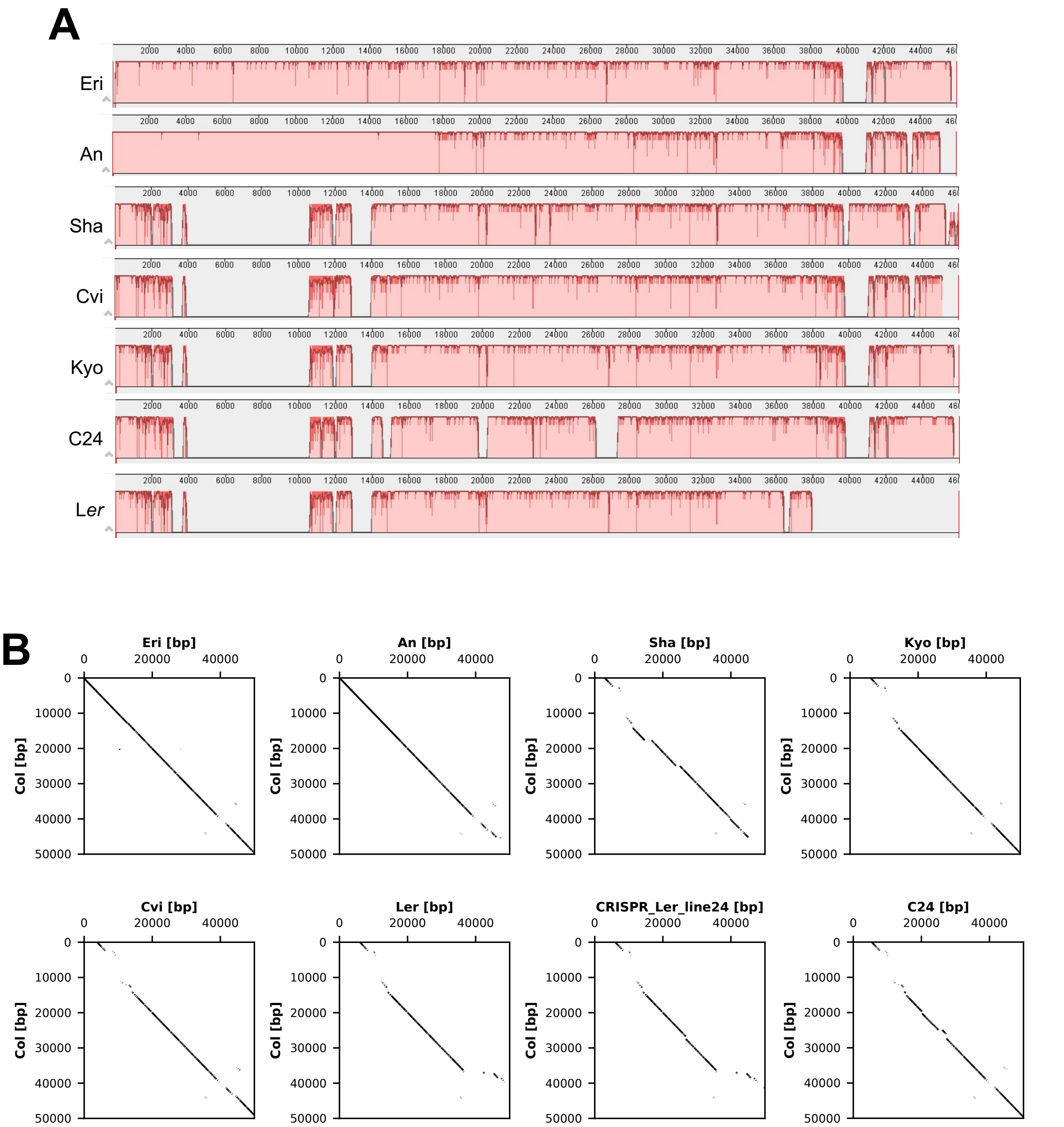

Supplementary Fig. 4. Structural variation between different *A. thaliana* accessions around Chili Pepper region. **A**, Genome alignment for a 46 kb region spanning the ChP interval (ChP 26 kb plus 10 kb from both sides) constructed using Mauve software [1]. Col sequence was used as a reference. Regions conserved between an accession and Col reference were shown in pink. **B**, Dotplots representing pairwise alignments for the ChP region (ChP 26 kb plus 10 kb from both sides) produced with Flexidot v.1.3 [2].

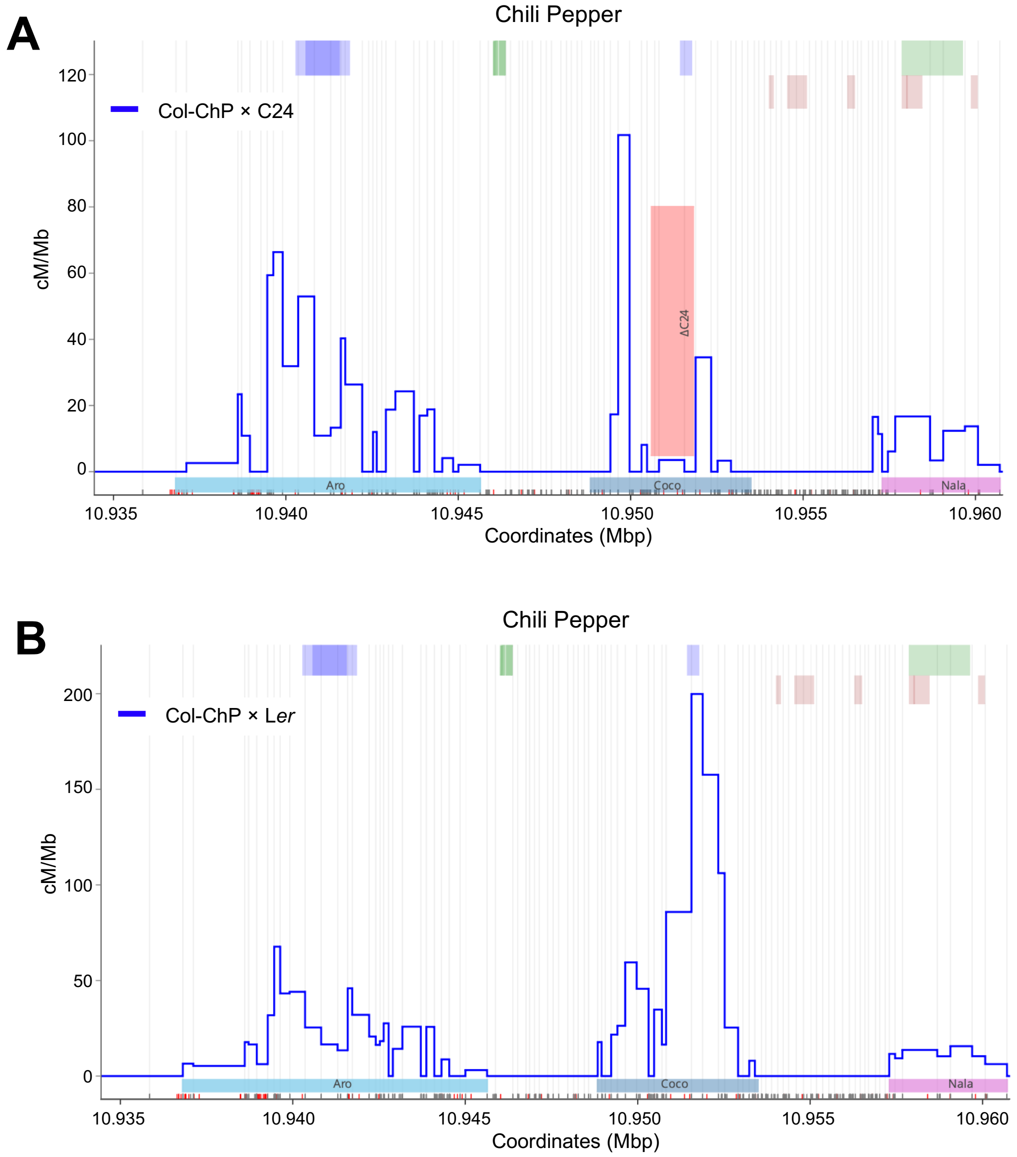

Supplementary Fig. 5. Crossovers in Col-ChP × C24 and Col-ChP × L*er*. **A**, CO landscape within ChP for a Col-ChP × C24 cross obtained by seed-typing. COs (blue lines) are normalized to the ChP CO frequency measured by seed scoring. SNPs spaced at least 100 bp apart, shown as vertical gray lines, were used to determine the CO topology. Black and red x-axis ticks correspond to all Col/C24 SNPs and InDels, respectively. Deletion within the Coco hotspot is indicated by the pink rectangle. Genes are shown as light-green (forward) and dark-green (reverse) rectangles, and transposons are shown as blue rectangles. Positions of three CO hotspots are indicated in colored rectangles below the plot. **B**, As in **A**, but for CO landscape within ChP for a Col-ChP × L*er* cross. Black and red x-axis ticks correspond to all Col/L*er* SNPs and InDels, respectively. Source data are provided as a Source Data file.

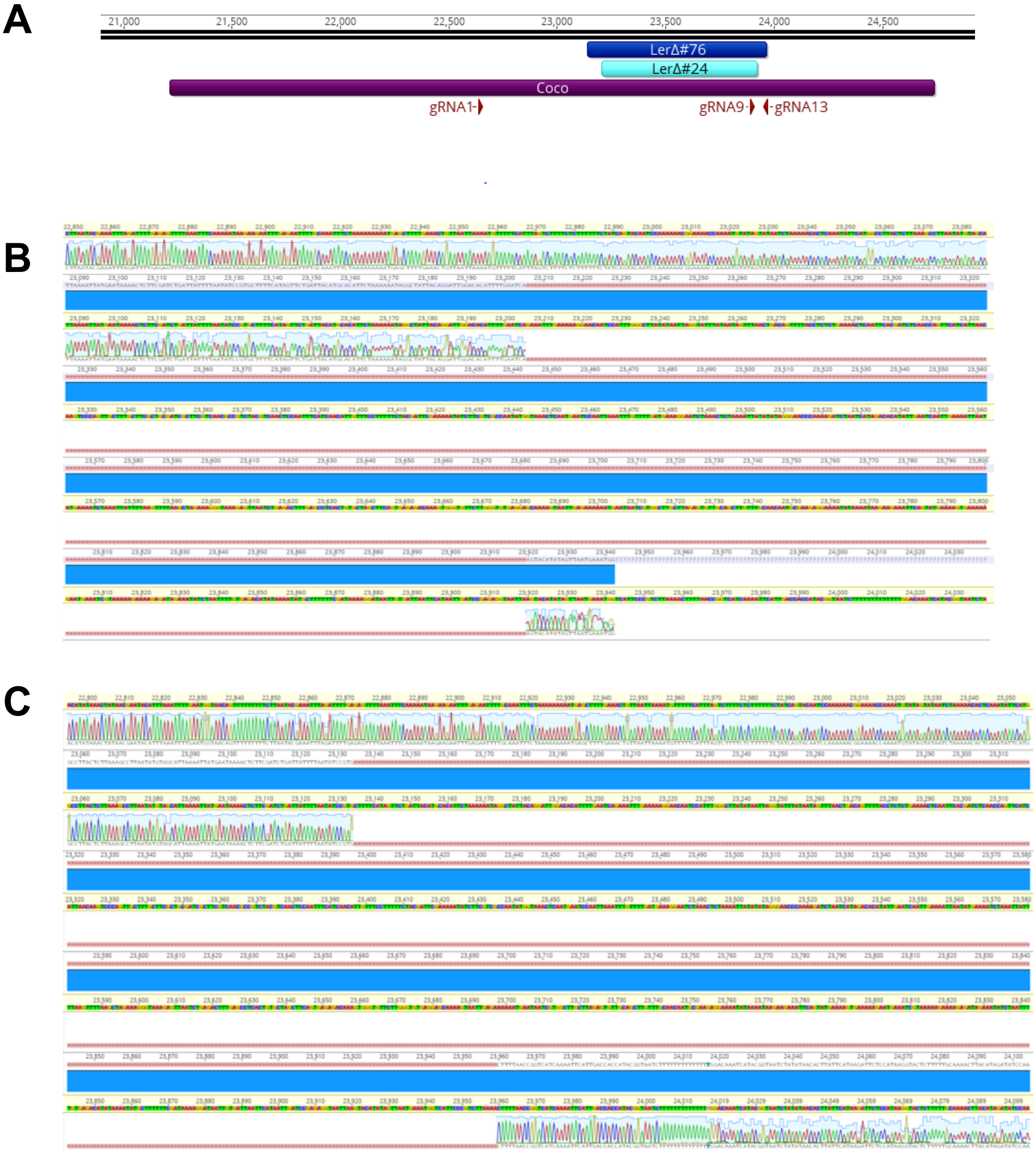

Supplementary Fig. 6. Cas9-generated deletion lines L*er*Δ#24 andL*er*Δ#76. **A,** *Coco* hotspot indicated in purple with deletions marked in dark blue (L*er*Δ#76) and light blue (L*er*Δ#24). gRNAs used to generate deletions are marked with red arrows (gRNA1 and gRNA9 for L*er*Δ#24 and gRNA1 and gRNA13 for L*er*Δ#76). **B**, Sequencing confirmation of 714 bp deletion in L*er*Δ#24 by alignment to L*er* reference. Deletion is marked with red dashes. **C**, Sequencing confirmation of 825 bp deletion in L*er*Δ#76 by alignment to L*er* reference. Deletion is marked with red dashes.

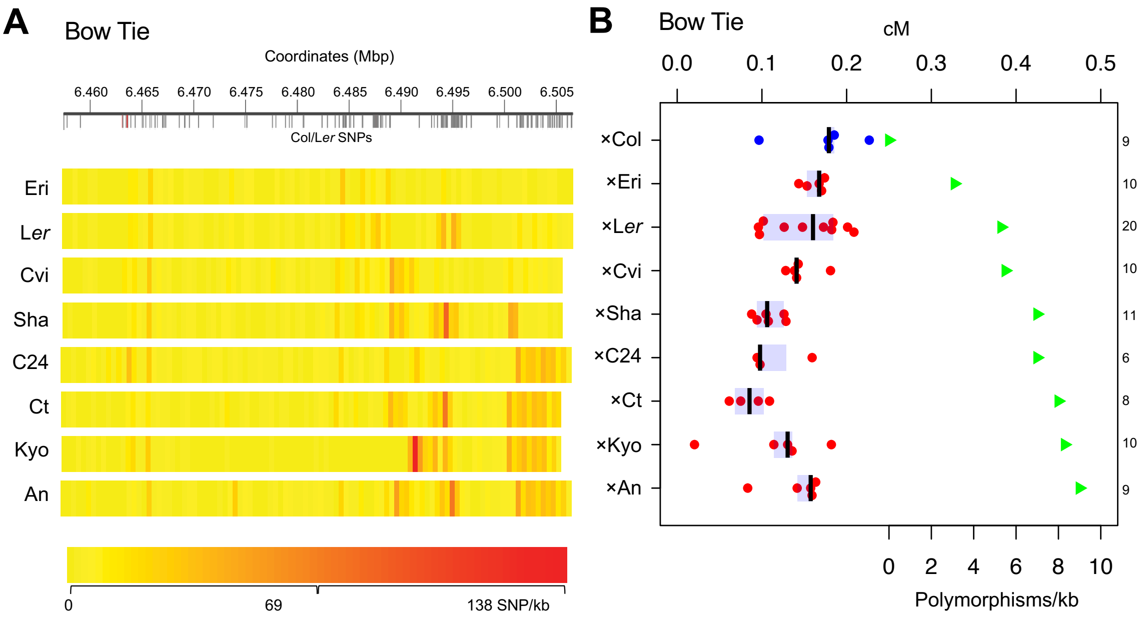

Supplementary Fig. 7. Effect of SNP on crossover frequency within the Bow Tie interval. **A**, SNP distribution within the BT interval in crosses between Col and different *A. thaliana* accessions. The SNP was estimated in 500 bp windows with color-scale shown below. **B**, Crossover frequency (cM) for different crosses between Col-BT and accessions shown in **A**. The center line of a boxplot indicates the mean; the upper and lower bounds indicate the 75th and 25th percentiles, respectively. Each dot represents a measurement from one or two pooled individuals. The numbers of individuals are indicted on the right from the boxplots. *P*-values indicate statistical difference of ChP CO rate in hybrids versus inbreds (Welch's *t* test). Green triangles indicate the polymorphism level (SNP+indels) for each cross within the BT interval. Source data are provided as a Source Data file.

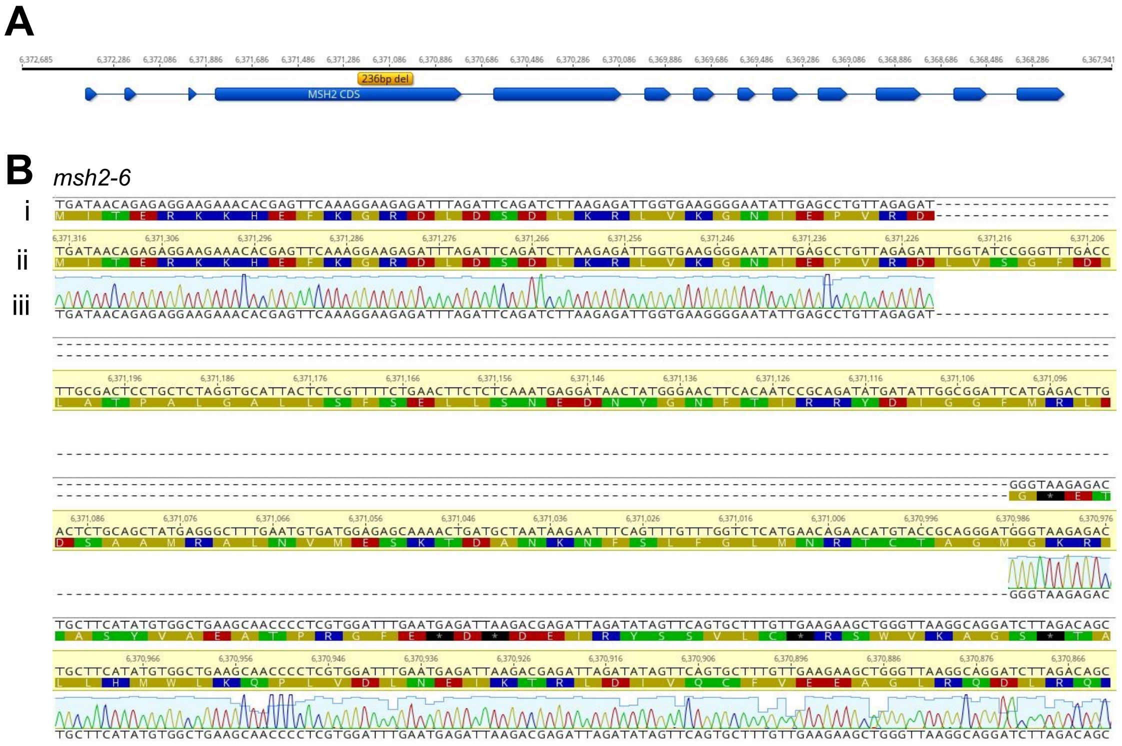

Supplementary Fig. 8. The *msh2* CRISPR/Cas9-mediated mutagenesis in L*er* accession. **A**, *MSH2* gene with exons represented as blue arrows and the CRISPR/Cas9 236bp deletion annotated in orange (4th exon). **B**, Sequence alignment of the L*er* *msh2-6* allele to the wildtype reference. i. DNA sequence and translation of the *msh2-6* 236bp deletion. ii. Wildtype reference. iii. Sequencing confirmation. *msh2-6* deletion introduces a frameshift and multiple STOP codons, represented in black.

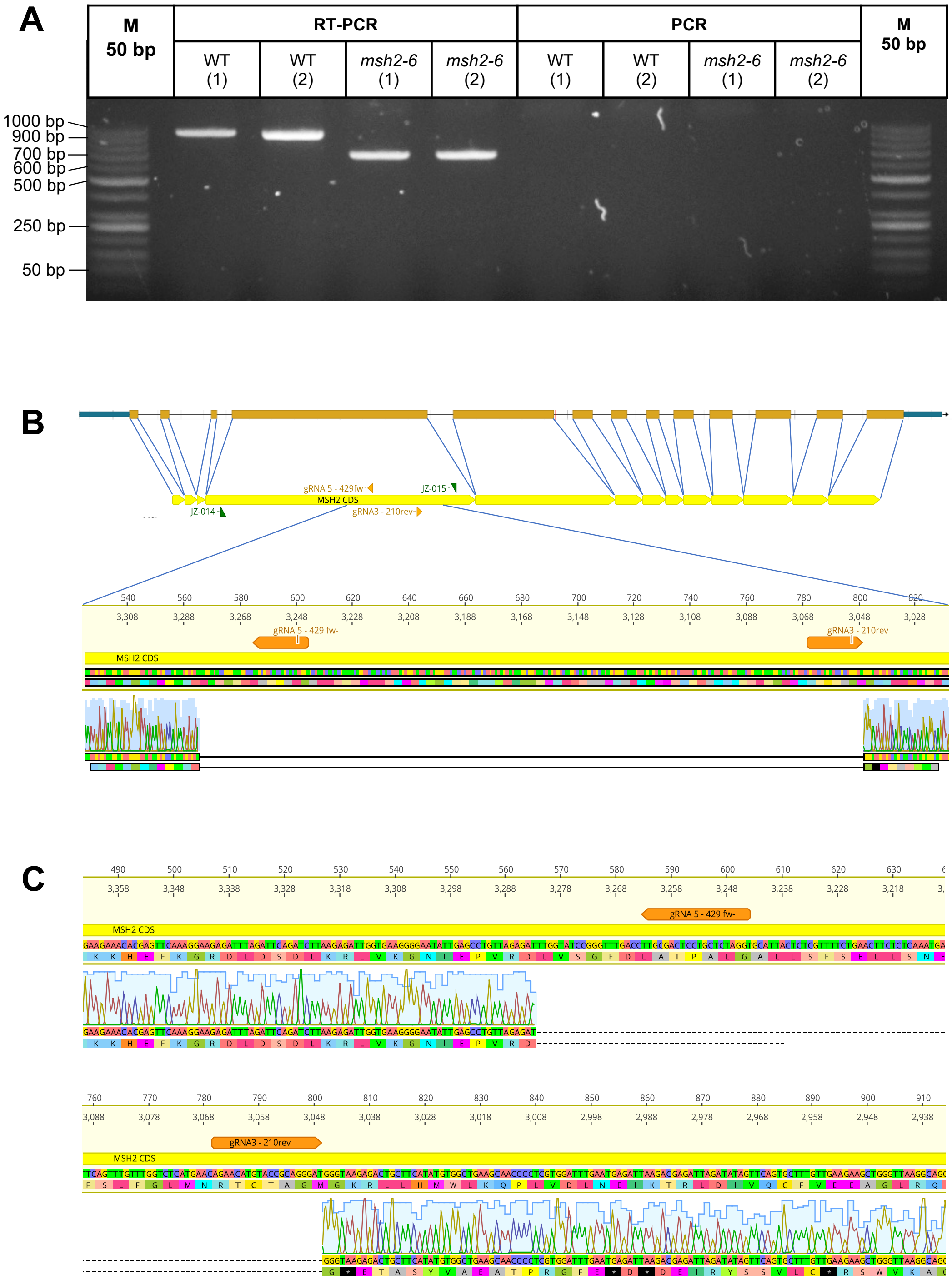

Supplementary Fig. 9. Verification of the *msh2-6* mutant allele (L*er* background) by RT-PCR. **A**, RT-PCR amplification of a transcript fragment flanking the deletion for wild type L*er* (WT) and the *msh2-6* mutant plants. Two independent samples were used for each genotype (number 1 and 2 indicated in parentheses). PCR was used as a control to rule out amplification from genomic DNA. **B**, Model of *MSH2* gene and the corresponding transcript with marked location of sgRNAs used to create the deletion (gRNA5 and gRNA3) and primers used for RT-PCR (JZ-014 and JZ-015). Shown is a fragment of a sequenced transcript for the *msh2-6* mutant. **C**, Sequence chromatogram of the *msh2-6* mutant transcript around the deletion site with the nucleotide and amino acid sequences indicated. Premature stop codons are highlighted in black. Source data are provided as a Source Data file.

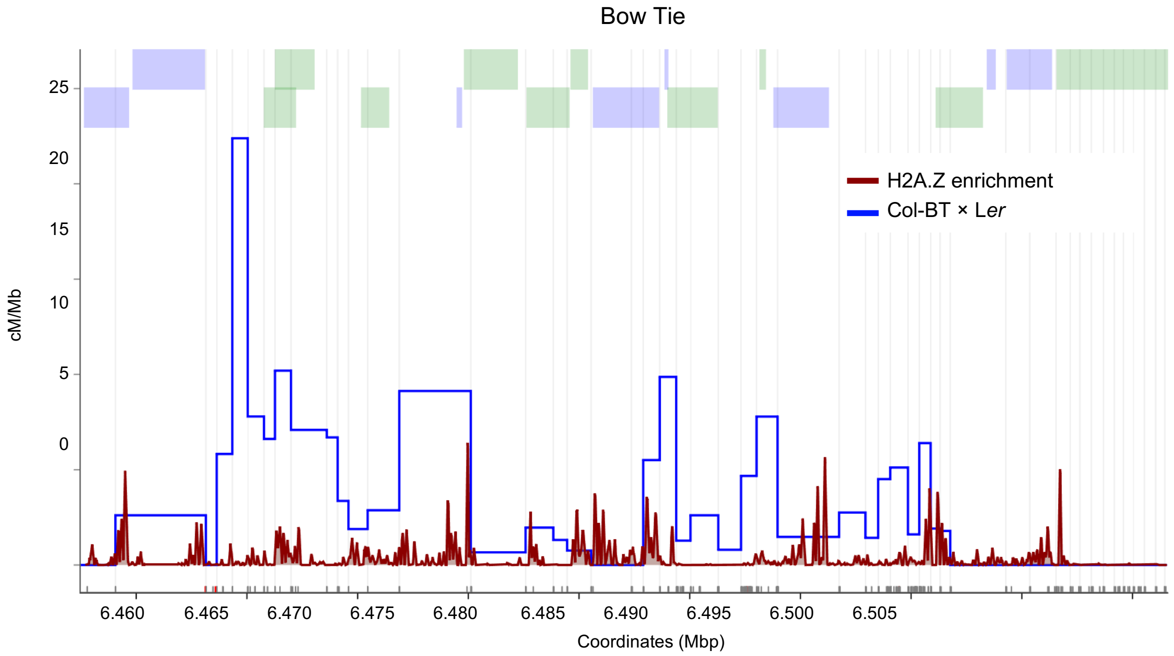

Supplementary Fig. 10. Crossover landscape within the Bow Tie interval for Col-BT × L*er* cross overlayed with a profile of H2A.Z histone variant used as a proxy for DSB sites. Crossovers (blue solid lines) are normalized to the number of sequenced recombinants and to the BT crossover frequency measured by seed scoring. H2A.Z profile [3] is shown as dark-red peaks. Black and red x-axis ticks correspond to Col/L*er* SNPs and indels, respectively. SNPs used to identify crossovers are shown also as vertical gray lines. Genes in forward and reverse orientation are presented as light-green and purple rectangles, respectively.

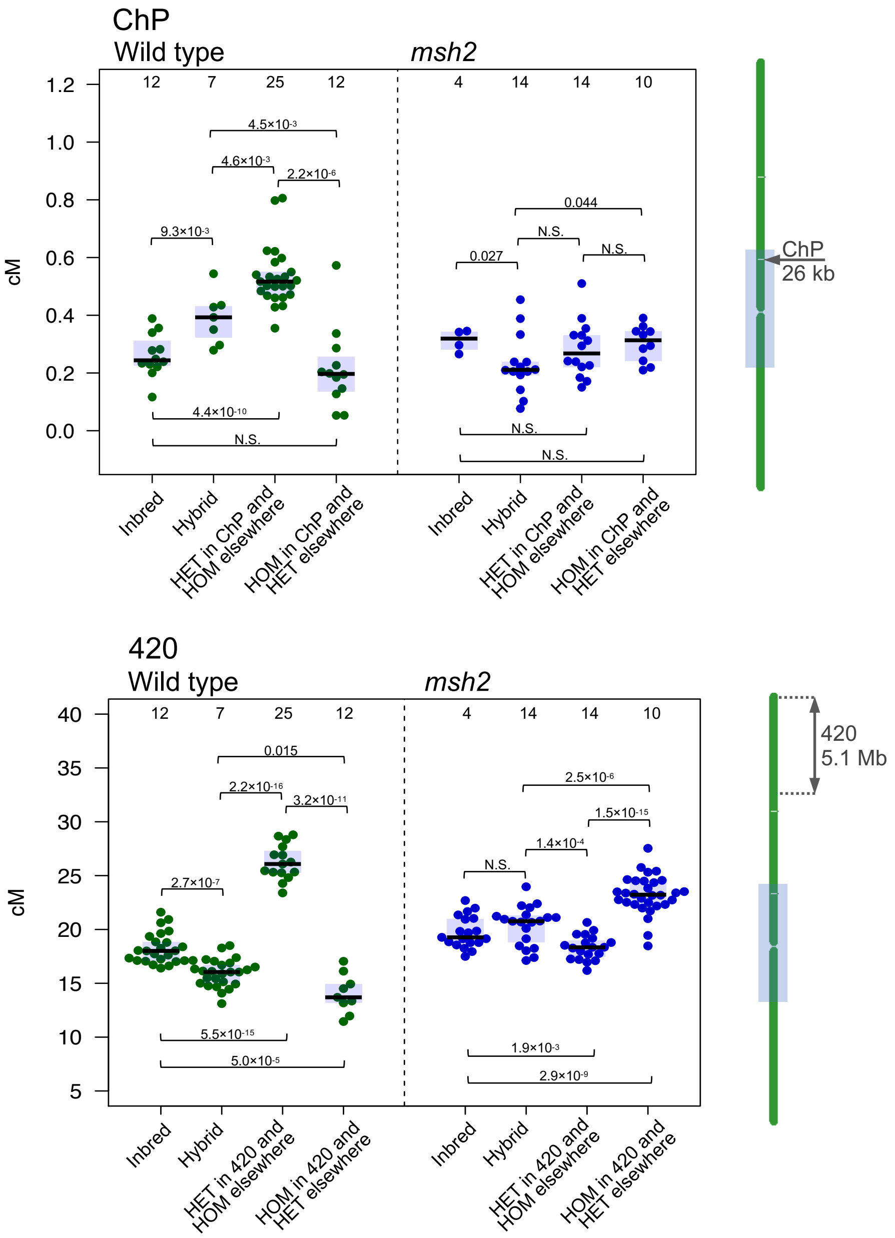

Supplementary Fig. 11. Heterozygosity-homozygosity juxtaposition effect at the kilobase scale. The center line of a boxplot indicates the mean; the upper and lower bounds indicate the 75th and 25th percentiles, respectively. Each dot represents a measurement from one individual. The numbers of individuals are also indicted below the boxplots. The two-sided *P*-values were estimated by Welch's *t* test without correction for multiple comparisons. The effects of the interhomolog polymorphism in the ChP interval (26 kb) resemble the effects observed at megabase scale in the 420 interval (5.1 Mb). In both cases, when the interval is heterozygous (HET) while the remainder of the genome is homozygous (HOM), the CO frequency in this interval is significantly higher than in the hybrid. When the interval is homozygous in an otherwise heterozygous genome, the CO frequency is significantly lower than in the hybrid. This effect disappears in the *msh2* mutant. The difference between ChP and 420 is in the comparison of inbreds vs. hybrids and is likely due to genetic differences between the Col/Col vs. Col/L*er* as previously observed [4]. A similar difference occurs when comparing ChP with BT (Fig. 5a, b). The data for ChP are the same as in Figure 8e while the data for 420 are from Blackwell et al.[5] Source data are provided as a Source Data file.

Supplementary Tables

Supplementary Table 1. Characteristics of the ESIL intervals.

ESIL	Size (bp)	Single-color reporter line**		Coordinates (bp)		Genetic size (cM)***		
		Left	Right	Left	Right	Col × Col	Col × L*er*	Col × Ct
Big Bertha (BB)	40175	CR1118	CG211	90,264	130,439	0.196	0.170 *N.S.*	0.310 *N.S.*
Bow Tie (BT)*	49116	CG10	CR874	6,457,489	6,506,605	0.173	0.152 *N.S.*	0.083 P=4.5×10^{-3}
Top Gun (TG)*	15161	CR874	CG670	6,506,605	6,521,766	0,007	0.030 *N.S.*	0.030 *N.S.*
Blind Fury (BF)*	32737	CG670	CR1375	6,521,766	6,554,503	0,185	0.095 P=0.024	0.172 *N.S.*
Chili Pepper (ChP)	26339	CG437	CR1004	10,934,456	10,960,795	0,239	0.480 P=1.1×10^{-4}	0.382 P=3.1×10^{-3}

* These ESILs are adjacent as they share reporters;

** Single-color reporter lines created by Wu et al.[6]

*** For Col × L*er* and Col × Ct hybrids, statistical difference is indicated when compared to Col × Col inbreds (two-sided Welch's *t* test), *N.S.*, not significant.

Supplementary Table 2. Polymorphism level within the ESIL intervals.

ESIL	Size (bp)	Ct × Col polymorphism		L*er* × Col polymorphism	
		**Total***	**SNPs only***	**Total**	**SNPs only**
Big Bertha (BB)	40175	3.91 (157)	2.91 (117)	4.88 (196)	3.78 (152)
Bow Tie (BT)	49116	8.02 (394)	6.29 (309)	4.97 (244)	3.58 (176)
Top Gun (TG)	15161	6.20 (94)	5.34 (81)	4.09 (62)	3.56 (54)
Blind Fury (BF)	32737	2.11 (69)	1.74 (57)	0.67 (22)	0.55 (18)
Chili Pepper (ChP)	26339	11.54 (304)	9.49 (250)	18.72 (493)	14.20 (374)

* Polymorphism density per kb; numbers per interval shown in parentheses.

Supplementary Table 3. Some features of the three hotspots in the ChP interval as analyzed in Col-ChP × L*er* cross

Hotspot	cM/Mb	Absolute CO rate (cM)	Left SNP (bp)	Right SNP (bp)	Size (bp)	Polymorphisms/kb (number)		
						SNPs	Indels	Total
Aro	17.23	0.152	10,936,812	10,945,633	8,821	15.87 (140)	4.19 (37)	20.07 (177)
Coco	62.14	0.288	10,948,841	10,953,480	4,639	11.64 (54)	3.02 (14)	14.64 (68)
Nala	11.57	0.039	10,957,305	10,960,705	3,400	5.00 (17)	1.76 (6)	6.76 (23)
Genome average	3.5*<br>3.2**					3.81		

*Based on Giraut et al. (2011)

**Based on Rowan et al. (2019)

Supplementary Table 4. Characteristics of the BT sections.

**Section**	**Section size (bp)**	**Wild type crossover frequency (cM/kb)**	***msh2* crossover frequency (cM/kb)**	**Relative crossover activity (100% × wild type / *msh2*)**	**Polymorphism density (SNP + indels/kb)**
1	5000	0.217	0.314	145	0.800
2	5200	0.767	0.942	123	7.353
3	3200	0.334	0.606	182	1.923
4	3200	0.634	0.987	156	1.875
5	3000	0.083	0.157	188	1.667
6	3000	0.083	0.112	135	2.000
7	2600	0.000	0.000	N/A	1.875
8	3400	0.317	0.359	113	6.346
9	3400	0.234	0.067	29	14.706
10	3200	0.150	0.651	433	0.625
11	4600	0.267	0.112	42	15.652
12	4800	0.000	0.090	N/A	2.917
13	4600	0.000	0.000	N/A	6.957

Supplementary Table 5. Characteristics of the R^2 lines.

R^2 line	Numbers of polymorphisms (indels + SNPs)	Numbers of SNPs	Coordinates of heterozygous region	
			Left	Right
R^2_1-1	293	244	10939460	10959696
R^2_1-4	280	232	10939909	10959696
R^2_1-6	280	232	10939909	10959696
R^2_2-31	155	129	10949175	10957673
R^2_2-33	44	34	10949175	10952447
R^2_2-37	44	34	10949175	10952447
R^2_2-36	44	34	10949175	10952447
R^2_2-28	40	31	10949969	10952447

Supplementary Table 6. Heterozygosity estimates for the R^2 lines.

R^2 line	Plant ID	Heterozygosity level (kb)						Percent of heterozygosity
		Chr. 1	Chr. 2	Chr. 3	Chr. 4	Chr. 5	Total	
R^2_1-1	2	9620	320505	494461	56195	550854	1431635	1.15
	4	4828145	8950551	4100318	1251857	903508	20034379	16.03
R^2_1-4	35	1805132	548571	2117966	1052653	828634	6352956	5.08
	41	2219763	1104418	2600420	2207471	493677	8625749	6.9
R^2_1-6	54	5689763	1250693	3780511	1869191	673398	13263556	10.61
	56	1934544	323824	2525656	1089096	1011255	6884375	5.51
R^2_1-1 *msh2*	73	1573072	1618475	3275531	1089045	965176	8521299	6.82
	74	3230924	1602800	1624945	1311050	368602	8138321	6.51
R^2_1-4 *msh2*	82	1443660	546265	494461	262303	32467	2779156	2.22
	83	2264480	1616255	2308889	953242	315525	7458391	5.97
R^2_2-33	117	2865581	329288	4234077	1056197	1379346	9864489	7.89
	118	5389474	977497	2071576	544642	6613649	15596838	12.48
R^2_2-28	140	1805132	1641191	498479	1056343	873883	5875028	4.7
	144	3868800	2015937	3217338	608023	1416043	11126141	8.9
R^2_2-37	151	1484889	2652709	1505554	398157	337583	6378892	5.1
	164	1424474	1544752	2098999	398157	1428790	6895172	5.51
R^2_2-31	169	1443660	326035	2118521	159150	100868	4148234	3.32
	173	1805132	398133	1610366	136875	834086	4784592	3.83
R^2_2-36	251	1443756	320505	609603	1055934	590796	4020594	3.22
	240	1805132	546587	1105936	953242	550881	4961778	3.97
R^2_2-33 *msh2*	183	854966	336591	4911829	953242	1103113	8159741	6.53
R^2_2-28 *msh2*	199	559030	512878	3695486	1056343	832521	6656258	5.3
	201	363947	320511	1115959	56195	100769	1957381	1.57
R^2_2-37 *msh2*	219	363947	1546801	2619192	1166799	828479	6525218	5.22
	223	3000548	3143846	1296630	192049	355264	7988337	6.39
R^2_2-31 *msh2*	232	1934544	546823	609850	262303	827895	4181415	3.35
	240	1934544	551671	2525656	398157	1010960	6420988	5.14

Supplementary Table 7. List of primers

ID	Primer sequence	Orientation	Comments
CR1118_LP	TGCAGCTGTTAACTGTAGGGTT	Forward	Multiplex with dsRed_F to genotype red cassette in BB
CR1118_RP	AATGGAAGATGTGGGTGGGG	Reverse	
CG211_LP	GGACCCTATCCGAGACTCCT	Forward	Multiplex with eGFP_F to genotype green cassette in BB
CG211_RP	AATCTGTTGAGGCCTTGGCA	Reverse	
CG670_LP	TGGGAACGAAACAGGGGAAA	Forward	Multiplex with eGFP_F to genotype green cassette in TG and BF
CG670_RP	AAACGGTTCCATGCTTTGCA	Reverse	
CR874_LP	TCTCCAGAGAGGTGTGCTAGT	Forward	Multiplex with dsRed_F to genotype red cassette in TG and BT
CR874_RP	CTGACTTTGCTGTGGCTTCA	Reverse	
CR1375_LP	TTCGGGCAAGGAGGGTAATG	Forward	Multiplex with dsRed_F to genotype red cassette in BF
CR1375_RP	TGATCAACCATGGCTTGCCT	Reverse	
CG10_LP	AAGGCAACAGAATCGCGTAA	Forward	Multiplex with eGFP_F to genotype green cassette in BT
CG10_RP	TATTTGCATGGGGAGAGCGT	Reverse	
CG437_LP	TTGATGGATCGAGACTTGTCG	Forward	Multiplex with eGFP_F to genotype green cassette in ChP
CG437_RP	TCAAACTCGACCGGAATTCCA	Reverse	
CR1004_LP	AGACGTACTAGCCGTGGAGT	Forward	Multiplex with dsRed_F to genotype red cassette in ChP
CR1004_RP	TGTTGGAGTTTGCCATTCATGA	Reverse	
eGFP_R	GAGCAAAGACCCCAACGAGA	Forward	
dsRed_F	GTATCCTCGTGATGGCGTGT	Forward	
ChP_1_Col, Ct	TGATAGTTCTCCGAAGAATACTTCCAT	Forward	LR-PCR of ChP Region 1 8787 bp in Col; 7477 bp in Ler
ChP_1_Ler, C24	GAAGGAAGGAGACAACTCTGATACT	Forward	
ChP_1_R	ACGACCTTCTTATTTGCCAATTCAT	Reverse	
ChP_2_F	GATTGGTTTAGCTGGTTGGATCCG	Forward	LR-PCR of ChP Region 2 10235 bp in Col
ChP_2_R	TACTTTTGCTCCTCACACCCAAGA	Reverse	
ChP_3_F	AGCTTCCTCTGCCACTAAATCACA	Forward	LR-PCR of ChP Region 3 10168 bp in Col
ChP_3_R	TTTTCAGACAAACTCCAATTTCACAG	Reverse	
BT1_F	CTCTCCCCATGCAAATATGACGTGTCAATA	Forward	LR-PCR of Region 1 in BT 10921 bp in Col
BT1_R	TAAGTTTTTGGTCCAACAAGAAGGCTTTGC	Reverse	
BT2_F	ATATCAAACTCCCCATCCTGAAACTGAAGC	Forward	LR-PCR of Region 2 in BT 10055 bp in Col
BT2_R	TCTCCTAATCCTTCGTTCTGAATCTCAGCT	Reverse	
BT3_F	GTCTTACTTTTGCTACCTCCATCTTCA	Forward	LR-PCR of Region 3 in BT 10970 bp in Col
BT3_R	AGCCATCGGATACAAAATGAAGATTAGCCT	Reverse	
BT4_F	ATATTTGCTTTCCCTTCGAGCTGATCTTGT	Forward	LR-PCR of Region 4 in BT 9625 bp in Col
BT4_R	TAACTGTGTCTCTCTCGAAGCTACTGTCAA	Reverse	
BT5_F	GGAAGATGGTGGAAGCGAAAAGGTTATTTG	Forward	LR-PCR of Region 5 in BT 9386 bp in Col
BT5_R	GTGTCCTTCAGTATATTAACGATTGCG	Reverse	
JZ-14	AACCACTGCTCTACGTCAGC	Forward	Genotyping and verification of msh2 mutations
JZ-15	TCGCTTCAGATGCTGTCTAA	Reverse	

Supplementary Table 8. Characteristics of sequence output for libraries of samples in which crossovers were identified.

Library name	Average Read Number	Average Read per SNP	Average read per kb	Number of individuals/ COs identified
Col-ChP × L*er*	383 877	1 561	14 596	243
Col-ChP × C24	563 267	1 969	21 417	177
Col-ChP × L*er*Δ#24	572 369	2 327	21 763	209
Col-ChP^{msh2} × Ler^{msh2}	598 867	2 444	22 771	187
Col-BT × L*er*	280 422	1 621	5 620	160
Col-BT^{msh2} × L*er*^{msh2}	200 321	1 185	4 014	196

Supplementary References

1. Darling, A. C. E., Mau, B., Blattner, F. R. & Perna, N. T. Mauve: multiple alignment of conserved genomic sequence with rearrangements. *Genome Res.* **14**, 1394–1403 (2004).
2. Seibt, K. M., Schmidt, T. & Heitkam, T. FlexiDot: highly customizable, ambiguity-aware dotplots for visual sequence analyses. *Bioinformatics* **34**, 3575—3577 (2018).
3. Sura, W. *et al.* Dual role of the histone variant H2A.Z in transcriptional regulation of stress-response genes. *Plant Cell* **29**, 791–807 (2017).
4. Lian, Q. *et al.* The megabase-scale crossover landscape is largely independent of sequence divergence. *Nat. Commun.* **13**, 3828 (2022).
5. Blackwell, A. R. *et al.* MSH2 shapes the meiotic crossover landscape in relation to interhomolog polymorphism in Arabidopsis. *EMBO J.* **39**, e104858 (2020).
6. Wu, G., Rossidivito, G., Hu, T., Berlyand, Y. & Poethig, R. S. Traffic lines: New tools for genetic analysis in Arabidopsis thaliana. *Genetics* **200**, 35–45 (2015).
7. Giraut, L. *et al.* Genome-wide crossover distribution in Arabidopsis thaliana meiosis reveals sex-specific patterns along chromosomes. *PLoS Genet.* **7**, e1002354 (2011).
8. Rowan, B. A. *et al.* An Ultra High-Density Arabidopsis thaliana Crossover. *Genetics* **213**, 771–787 (2019).
