# Supplementary material for: The effect of DNA polymorphisms and natural variation on crossover hotspot activity in Arabidopsis hybrids
Source: Nat Commun. 2023 Jan 3;14:33. doi: 10.1038/s41467-022-35722-3 (PMC9810609; doi:10.1038/s41467-022-35722-3)
Supplement: Supplementary file 3 — Description of Additional Supplementary Files [file 41467_2022_35722_MOESM3_ESM.pdf]

Description of Additional Supplementary Files

File Name: Supplementary Data 1
Description: Raw data for crossover frequency measurements by seed scoring.
