# Supplementary material for: The effect of DNA polymorphisms and natural variation on crossover hotspot activity in Arabidopsis hybrids
Source: Nat Commun. 2023 Jan 3;14:33. doi: 10.1038/s41467-022-35722-3 (PMC9810609; doi:10.1038/s41467-022-35722-3)
Supplement: Supplementary file 5 — Reporting Summary [file 41467_2022_35722_MOESM5_ESM.pdf]

nature portfolio

Corresponding author(s): Piotr A. Ziolkowski

Last updated by author(s): Dec 20, 2022

Reporting Summary

Nature Portfolio wishes to improve the reproducibility of the work that we publish. This form provides structure for consistency and transparency in reporting. For further information on Nature Portfolio policies, see our Editorial Policies and the Editorial Policy Checklist.

Statistics

For all statistical analyses, confirm that the following items are present in the figure legend, table legend, main text, or Methods section.

n/a	Confirmed	
☐	☒	The exact sample size (*n*) for each experimental group/condition, given as a discrete number and unit of measurement
☐	☒	A statement on whether measurements were taken from distinct samples or whether the same sample was measured repeatedly
☐	☒	The statistical test(s) used AND whether they are one- or two-sided<br>*Only common tests should be described solely by name; describe more complex techniques in the Methods section.*
☐	☒	A description of all covariates tested
☐	☒	A description of any assumptions or corrections, such as tests of normality and adjustment for multiple comparisons
☐	☒	A full description of the statistical parameters including central tendency (e.g. means) or other basic estimates (e.g. regression coefficient) AND variation (e.g. standard deviation) or associated estimates of uncertainty (e.g. confidence intervals)
☐	☒	For null hypothesis testing, the test statistic (e.g. *F*, *t*, *r*) with confidence intervals, effect sizes, degrees of freedom and *P* value noted<br>*Give P values as exact values whenever suitable.*
☒	☐	For Bayesian analysis, information on the choice of priors and Markov chain Monte Carlo settings
☒	☐	For hierarchical and complex designs, identification of the appropriate level for tests and full reporting of outcomes
☒	☐	Estimates of effect sizes (e.g. Cohen's *d*, Pearson's *r*), indicating how they were calculated

Our web collection on statistics for biologists contains articles on many of the points above.

Software and code

Policy information about availability of computer code

Data collection	The related code is available at GitHub [https://github.com/LabGenBiol/ESILs]
Data analysis	Identification of crossover breakpoints<br>To identify SNPs within the ChP or BT intervals between Col and other Arabidopsis accessions (Ler, C24), demultiplexed paired-end forward and reverse reads have been pooled and aligned to Col reference sequence with use of bwa-mem algorithm from Burrows-Wheeler Aligner (BWA) software v0.7.17-r1188 (Li and Durbin, 2009). Resulting BAM files have been sorted and indexed with use of SAMtools v1.2 (Li et al., 2018). SNPs were called using SAMtools and BCFtools v1.2 (Li, 2011). Subsequently, SNP with low coverage (<30 reads) and poor quality have been filtered out from the list. Individual sequencing libraries have been aligned to Col-0 ChP or BT sequences with use of bwa-mem algorithm with use of default parameters and compared to previously generated SNP list with SAMtools and BCFtools. Custom R script (https://www.r-project.org) enabled comparison of the percentage of reads associated with reference (Col) or variant nucleotides in Ler and C24 accessions, and are available from the authors on request. This has further allowed for genotype determination at particular SNP location and manual designation of CO breakpoint.

For manuscripts utilizing custom algorithms or software that are central to the research but not yet described in published literature, software must be made available to editors and reviewers. We strongly encourage code deposition in a community repository (e.g. GitHub). See the Nature Portfolio guidelines for submitting code & software for further information.

Data

Policy information about availability of data

All manuscripts must include a data availability statement. This statement should provide the following information, where applicable:

- Accession codes, unique identifiers, or web links for publicly available datasets
- A description of any restrictions on data availability
- For clinical datasets or third party data, please ensure that the statement adheres to our policy

All data generated or analyzed during this study are included in this published article (and its supplementary information files). Raw data related to the seed-typing sequencing (WCS-seq) are deposited in NCBI Sequence Read Archive (SRA), BioProject PRJNA882919 [https://dataview.ncbi.nlm.nih.gov/object/PRJNA882919]. The Col-0 TAIR10 reference genome is downloaded from the TAIR database [https://www.arabidopsis.org/]. Genomic sequences of Arabidopsis thaliana accessions (An-1, C24, Cvi-0, Eri-1, Kyo, Ler, Sha) used in this study can be downloaded from https://1001genomes.org/projects/MPIPZJiao2020/index.html]. Col × Ler F2 high density crossover data is downloaded from FigShare [https://doi.org/10.25386/genetics.9733838].

Human research participants

Policy information about studies involving human research participants and Sex and Gender in Research.

Reporting on sex and gender	N/A
Population characteristics	N/A
Recruitment	N/A
Ethics oversight	N/A

Note that full information on the approval of the study protocol must also be provided in the manuscript.

Field-specific reporting

Please select the one below that is the best fit for your research. If you are not sure, read the appropriate sections before making your selection.

☒ Life sciences ☐ Behavioural & social sciences ☐ Ecological, evolutionary & environmental sciences

For a reference copy of the document with all sections, see nature.com/documents/nr-reporting-summary-flat.pdf

Life sciences study design

All studies must disclose on these points even when the disclosure is negative.

Sample size	Recombination frequency (RF) was measured in the investigated Extremely Short Interval Lines (ESILs) using seed-based system, which was already described (Kbiri et al., 2022; Ziolkowski et al., 2015). The RF is presented in cM or cM/Mb and was calculated as a percent of single-colour recombinant seeds divided by the total number of seeds derived from F1 plants obtained after crossing ESILs with the non-colour accessions. RF for each genotype was calculated using 12,000 to 140,000 total number of seeds.<br>Crossover breakpoints were identified based on libraries sent for sequencing: 243 samples from F2 Ler × Col-ChP population, 177 samples from F2 C24 × Col-ChP population, 209 samples from F2 Col-ChP × LerΔ#24 population, 187 samples from F2 Ler msh2 × Col-ChP msh2 population, 160 samples from F2 Ler × Col-BT population, 196 samples from F2 Ler msh2 × Col-BT msh2 population. The sample size for crossover breakpoints in the ChP interval was estimated to obtain 6 to 10 mapped crossovers per 1 kb, per genotype, e.g., 6 COs × 26 kb = 156 samples. Such a resolution provides reliable data, as it is at least 500× higher than genome-wide CO maps constructed based on standard genotyping-by-sequencing method. In case of the BT interval, which has significantly lower SNP density (3.8 SNPs per 1 kb), the sample size was estimated to obtain between 3 to 6 mapped crossovers per 1 kb, per genotype. This sample size is sufficient with such low SNP density to provide reliable data. The resolution obtained for the BT interval is more than 260× higher than in genome-wide CO maps.
Data exclusions	No data were excluded.
Replication	For recombination frequency measurements each biological replicate corresponds to at least 1000 seeds derived from one F1 plant. At least five biological replicated were used for almost all measurements with the exception for Ct-Col-TG (3 biological replicates) and Ct-Col-BF (4 biological replicates). The number of replicates were counted to represent at least 12,000 seeds per genotype, the average number is above 40,000 events per genotype. All attempts at replication were successful.
Randomization	The plants used for RF measurements and crossover breakpoint identification were always grown in the same growth chamber with the same growth conditions. For each experiment, tested and control plants were randomly allocated in the growth area. For each experiment, respective control plants were grown along side the tested plants.
Blinding	In case of crossover breakpoints blinding is not necessary as the identification is based on SNP location which is objective. In case of crossover

Blinding	frequency measurements, samples were single blind.

Reporting for specific materials, systems and methods

We require information from authors about some types of materials, experimental systems and methods used in many studies. Here, indicate whether each material, system or method listed is relevant to your study. If you are not sure if a list item applies to your research, read the appropriate section before selecting a response.

Materials & experimental systems

n/a	Involved in the study
☒	☐ Antibodies
☒	☐ Eukaryotic cell lines
☒	☐ Palaeontology and archaeology
☐	☒ Animals and other organisms
☒	☐ Clinical data
☒	☐ Dual use research of concern

Methods

n/a	Involved in the study
☒	☐ ChIP-seq
☒	☐ Flow cytometry
☒	☐ MRI-based neuroimaging

Animals and other research organisms

Policy information about studies involving animals; ARRIVE guidelines recommended for reporting animal research, and Sex and Gender in Research

Laboratory animals	The study did not involve laboratory animals but laboratory plants
Wild animals	The study did not involve wild animals
Reporting on sex	*Indicate if findings apply to only one sex; describe whether sex was considered in study design, methods used for assigning sex. Provide data disaggregated for sex where this information has been collected in the source data as appropriate; provide overall numbers in this Reporting Summary. Please state if this information has not been collected. Report sex-based analyses where performed, justify reasons for lack of sex-based analysis.*
Field-collected samples	The study did not involve samples collected from the field
Ethics oversight	*Identify the organization(s) that approved or provided guidance on the study protocol, OR state that no ethical approval or guidance was required and explain why not.*

Note that full information on the approval of the study protocol must also be provided in the manuscript.
